# The protocol of the Oslo Study of Clonidine in Elderly Patients with Delirium; LUCID: a randomised placebo-controlled trial

**DOI:** 10.1186/s12877-015-0006-3

**Published:** 2015-02-10

**Authors:** Bjørn Erik Neerland, Karen Roksund Hov, Vegard Bruun Wyller, Eirik Qvigstad, Eva Skovlund, Alasdair MJ MacLullich, Torgeir Bruun Wyller

**Affiliations:** 1 Oslo Delirium Research Group, Department of Geriatric Medicine, University of Oslo, Pb 4956, N-0424, Oslo, Norway; 2 Department of Geriatric Medicine, Oslo University Hospital, Oslo, Norway; 3 Department of Pediatrics, Akershus University Hospital, Lørenskog, Norway; 4 Institute of Clinical Medicine, University of Oslo, Oslo, Norway; 5 Department of Cardiology, Oslo University Hospital, Oslo, Norway; 6 School of Pharmacy, University of Oslo, Oslo, Norway; 7 Edinburgh Delirium Research Group, Geriatric Medicine, University of Edinburgh, Room F1424, Royal Infirmary of Edinburgh, 51 Little France Crescent, Edinburgh, EH16 4SA UK

**Keywords:** Delirium, Treatment, Clonidine, Drug therapy, Double-blind method, Aged, Therapeutic use, Adrenergic alpha-2 Receptor Agonists

## Abstract

**Background:**

Delirium affects 15% of hospitalised patients and is linked with poor outcomes, yet few pharmacological treatment options exist. One hypothesis is that delirium may in part result from exaggerated and/or prolonged stress responses. Dexmedetomidine, a parenterally-administered alpha2-adrenergic receptor agonist which attenuates sympathetic nervous system activity, shows promise as treatment in ICU delirium. Clonidine exhibits similar pharmacodynamic properties and can be administered orally. We therefore wish to explore possible effects of clonidine upon the duration and severity of delirium in general medical inpatients.

**Methods/Design:**

The Oslo Study of Clonidine in Elderly Patients with Delirium (LUCID) is a randomised, placebo-controlled, double-blinded, parallel group study with 4-month prospective follow-up. We will recruit 100 older medical inpatients with delirium or subsyndromal delirium in the acute geriatric ward. Participants will be randomised to oral clonidine or placebo until delirium free for 2 days (Diagnostic and Statistical Manual of Mental Disorders (DSM-5) criteria), or after a maximum of 7 days treatment. Assessment of haemodynamics (blood pressure, heart rate and electrocardiogram) and delirium will be performed daily until discharge or a maximum of 7 days after end of treatment. The primary endpoint is the trajectory of delirium over time (measured by Memorial Delirium Assessment Scale). Secondary endpoints include the duration of delirium, use of rescue medication for delirium, pharmacokinetics and pharmacodynamics of clonidine, cognitive function after 4 months, length of hospital stay and need for institutionalisation.

**Discussion:**

LUCID will explore the efficacy and safety of clonidine for delirium in older medical inpatients.

**Trial registration:**

ClinicalTrials.gov NCT01956604. EudraCT Number: 2013-000815-26

## Background

### Delirium in hospitalised medical patients

Delirium commonly affects older people with acute medical illness [1,2]. It is associated with patient distress, increased length of stay, higher risk of new institutionalisation, higher mortality, and an increased risk of future dementia [3-6]. Despite its impact, delirium remains poorly understood and few treatment options are available.

The prevalence of delirium is at least 15% in hospitalised medical patients [7-9]. In patients with dementia the prevalence is even higher, being up to 50 % in medical wards [10]. Old age and pre-existing cognitive impairment are the most important predisposing factors for delirium [1,9], but, in principle, any acute medical condition can precipitate delirium in a vulnerable individual [7]. Subsyndromal delirium is a clinical condition that falls on a continuum between no symptoms and delirium defined by the Diagnostic and Statistical Manual of Mental Disorders-5 (DSM-5) [11]. Subsyndromal delirium may progress to full-scale delirium, and is a clinically important condition associated with poor outcomes. Efforts to prevent, detect and treat subsyndromal delirium are thus justified.

### Pathophysiology of delirium

The pathophysiology of delirium remains poorly understood. Leading hypotheses focus on neurotransmission, inflammation and acute stress as possible mechanisms [12]. Decreased cholinergic activity and increased dopaminergic activity in the central nervous system (CNS) are commonly postulated, with these neurotransmitter deficits resulting from multiple precipitants. A causal association between inflammation (pro-inflammatory cytokines) and delirium has also been proposed [13,14]. Another hypothesis implies that delirium may be the result of aberrant stress responses [13] as delirium commonly follows stress (physical or psychological). And whilst stress responses are adaptive in young people, they might be altered with ageing, leading to exaggerated and more prolonged stress responses. This hypothesis would also imply alterations of autonomic nervous system activity in patients with delirium [15].

### Pharmacological treatment of delirium

The hyperactive form of delirium is frequently treated pharmacologically, but the effect of different treatment modalities is poorly studied. The drug treatment of hypoactive delirium has hardly been evaluated at all. Thus more research on pharmacological treatment options for delirium is urgently needed. Haloperidol [16,17] and other antipsychotics (olanzapine [18], risperidone [19] and quetiapine [20]) are commonly used, and there is some, although weak, evidence of their efficacy [21,22]. There is no evidence on the effects of antipsychotics in patients with delirium superimposed upon dementia. For patients with parkinsonism or dementia with Lewy bodies, antipsychotics are generally avoided, leaving even fewer treatment options for these patients.

Antipsychotics have several drawbacks, including an increased risk of death and cardiovascular events, sedation, falls risk, and cognitive impairment [23]. These risks increase with age, dementia and disability. This calls for special caution for patients with delirium superimposed on dementia [21]. Benzodiazepines and other sedatives are also frequently used, but the evidence supporting these agents is even weaker [24]. There is no clear evidence for the efficacy of cholinesterase inhibitors in the treatment of delirium [25-27].

### Alpha-2-adrenoreceptor agonists

Dexmedetomidine and clonidine are both alpha-2-adrenoceptor agonists activating presynaptic inhibitory alpha-2-adrenoreceptors. Clonidine is a partial agonist with an alpha-2a-to-alpha-1 selectivity ratio of 39. For dexmedetomidine this ratio is 1300 [28]. They exert a general inhibitory influence on the sympathetic nervous system, in particular due to CNS effects [29]. Dexmedetomidine decreases sympathetic activity and attenuates the hemodynamic and neuroendocrinal stress responses (including of cortisol), resulting in decreased heart rate (HR) and blood pressure (BP) as well as sedative and analgesic effects [30]. In the intensive care setting, both substances reduce the need for opioids and sedatives [31]. Dexmedetomidine is increasingly used in intensive care patients, and is also used as an adjuvant during regional anaesthesia. Recently conducted studies indicate that the incidence of delirium is lower in intensive care patients receiving dexmedetomidine than in those receiving benzodiazepines [32,33], propofol [34] or morphine [35]. Currently available evidence thus suggests that dexmedetomidine may have value in the prevention and treatment of delirium in the intensive care unit [36,37]. Indeed, it is now in clinical use in the USA and Europe [38].

Most patients with delirium are however treated outside of intensive care units, where intravenous use of dexmedetomidine is not feasible. An alternative agent might thus be orally administred clonidine, which has very similar pharmacodynamics to that of dexmedetomidine [31], even though its alpha-2 selectivity is somewhat lower [28]. Clonidine additionally has an independent stimulatory effect on the parasympathetic activity and a slight anti-inflammatory effect [39,40], further making it an interesting candidate for delirium treatment. This does also accord with the hypothesis of delirium as a consequence of aberrant stress responses [13].

### Clinical experience with clonidine

Clonidine has been used as an anti-hypertensive drug for decades, as well as for anesthesia-related applications, such as perioperative analgesia [29,41], sedation and anxiolysis, and for management of both acute postoperative, chronic and neuropathic pain [41-43]. In one study, use of intravenous clonidine after surgical correction of acute type-A aortic dissection reduced the severity of delirium, improved the respiratory function and shortened the length of stay in the intensive care unit [44].

The adverse effects of clonidine include orthostatic hypotension, bradycardia and AV-block. Such effects are, however, dependent on dosage. In a previous report, low-dose treatment (75 μg) in healthy adults was associated with a reduction in heart rate from 72 beats/min to 63 beats/min, and a reduction in mean arterial pressure (MAP) from 88 mmHg to 75 mmHg. Less than 50% experienced sedation, dry mouth or dizziness, and for those who reported any of these side-effects, the severity was mild [45]. Clonidine has been studied in outpatients with Alzheimer's dementia [46,47] and in patients with Parkinson's disease [48,49]. Treatment with daily doses of clonidine less than 200 mcg was well tolerated in these patients. Relative contraindications to clonidine for its licensed indications include bradyarrhythmias, polyneuropathy, renal insufficiency and evidence of reduced cerebral and/or peripheral circulation due to vessel disease

### Pharmacokinetics of clonidine

Maximum plasma concentration (Cmax) following oral administration occurs after 1–3 h [45] and reduction in mean arterial pressure as well as the risk of side effects is highest at this peak. Cmax and area under the concentration-time-curve (AUC) increase proportionally with increasing doses. Clonidine traverses the blood–brain-barrier. The half-life during the elimination phase shows great inter-individual variation and is found to be between 5 and 25.5 hours. The metabolism is hepatic and the metabolites are inactive. Clonidine is mainly excreted renally (70%).

### Rationale for dosage plan of clonidine

A dosage plan for clonidine is presented in Table 1. Because we are studying short-term use in an acute setting, we want to achieve steady-state (serum concentration levels) more quickly by using loading doses (day 1) under close monitoring of blood pressure. On days 2–7 we will administer a lower maintenance dose. We will thus give 75 μg clonidine every 3^rd^ hour up to a maximum of 4 doses on day 1, and then 75 μg twice per day.

**Table 1:** **Dosage plan for clonidine**

Time	Safety	Dosage
Day 1	Systolic BP has to be >120 mmHg before the first loading dose.	75 μg every 3^rd^ hour until maximum 4 doses, (e.g.: at 2, 5, 8 and 11 p.m)
Loading doses	If systolic BP is <100 mmHg, HR <50 beats/min, or if RASS is −3 or less before any of the subsequent loading doses, no more study medication will be given until the planned maintenance dose the next morning.	
	If RASS is −2, the treating physician has to assess if IMP will be given or not	
Day 2-7	If systolic BP is <100 mmHg, HR <50 beats/min, or if RASS is −3 or less just before a planned dose, no study medication will be given until the next planned dose 12 hours later.	75 μg BID, at 8–9 a.m and 8–9 p.m
Maintenance doses		
	If RASS is −2, the treating physician has to assess if IMP will be given or not	

Concentration levels of clonidine known to have clinical effects range from 0.2 to 2.0 ng/ml [50-52]. We are aiming for the lower levels, that is, between 0.3 ng/ml (median trough concentration) and 0.7 ng/ml (maximal concentration), because higher plasma concentration levels increases the risk of adverse events, including hypotension. Nonetheless, lower plasma concentration levels may be insufficient to give a significant effect on our primary endpoint.

To our knowledge, there are no studies of the relationship between plasma concentration of clonidine and delirium. In publications on the effect on delirium of dexmedetomidine, the plasma concentration levels are not reported. Additionally, dexmedetomidine is approximately 8 times more selective (alpha 2 versus alpha 1) than clonidine [28,31], and so pharmacokinetic data on dexmedetomidine cannot be used to estimate effective doses of clonidine. We thus have to extrapolate from pharmacokinetic data on clonidine used for other purposes. We know that clonidine has both sedative and anxiolytic effects, and that these centrally mediated effects are closely related to plasma concentration levels [53,54].

In a study of adolescents with chronic fatigue syndrome, a dosage of 50 μg twice per day resulted in a median trough concentration (C_0_) at 0.21 μg/L after 14 days of treatment, rising to a median level of 0.41 μg/L (C_max_) two hours after administration of one regular dose of 50 μg [55,56]. In a study of healthy normotensive subjects, treatment with oral clonidine 225 μg daily for one week resulted in a steady state of 0.3-0.35 ng/ml. After intake of one 75 μg tablet, the serum level then increased to 0.7 ng/ml at 2 h. There was a significant relationship between the plasma level of clonidine and sedation [53]. In subjects receiving oral clonidine 100 μg twice per day for 6 weeks, plasma concentration ranged between 0.4 and 0.7 ng/ml (levels 2 hours after intake of 100 μg) [52]. Another study found that a single dose of 75 μg gave a Cmax of 0.66 ng/ml after achieving steady-state with two 75 μg doses [57].

The present patient population (elderly with a glomerular filtration rate [GFR] limit of > 30 ml/min) may have a longer half life of clonidine due to a diminished renal capacity as compared to younger. Altough the metabolism of clonidine is hepatic, varying amounts of unmetabolised clonidine is secreted renally. Consequently, patients may also have a higher risk of adverse events related to the study drug. We therefore choose a lower dosage, considered safe, of 75 μg twice per day.

### Loading doses

The loading dose is based on half-life. Clonidine follows first order kinetics for elimination, and thus the mean steady state is proportional to the daily dose. Doubling the total daily dose should double the mean plasma concentration level. A study showed that a single oral dose of 75ug clonidine gave Cmax 0.29 ng/ml, 150ug gave Cmax 0.61 ng/ml and 250ug gave 1.2 ng/ml [45]. We will administer up to 300 μg the first day, but as 75 ug every three hours. Thus, the theoretical Cmax at day one would be significantly lower than 1.2 ng/ml, due to elimination of the drug. How much lower is difficult to estimate, given the large variability in half-life (5 to 25 hours).

The expected maximum hypotensive effect correlates well with the Cmax time-point occurring after 2 hours. There is an interindividual difference in the pharmacodynamic response to clonidine. Our loading dose is dependent on and monitored by the individual patients' haemodynamic response (blood pressure and heart rate).

### Safety review

A safety review will be done after we have recruited 20 patients (10 placebo and 10 clonidine). Recruitment of the additional 80 patients will be halted until we have made an assessment of the serum concentration levels, pharmacokinetics and -dynamics (the haemodynamic response) in these initial 20 patients. These data will then guide further dosage for the subsequent 80 patients. We are aiming for serum concentration levels between 0.3 and 0.7 ng/ml, but may accept a small number of single measures above this range. Blood samples for serum concentration measurement will be taken 3 hours after the patient has taken the study drug (each time) on day 1 and just before administration of study drug between 0800 and 0900 on day 2. The serum concentration levels and pharmacodynamic responses will be assessed by an independent Data Monitoring Committee. The determination of the further dosage plan will thus be based on both pharmacokinetics/serum concentration levels and the individual patients´ haemodynamic responses. The further dosage plan will be approved by The Norwegian Medicines Agency.

## Study objectives

### Primary endpoint

The endpoints and measurements for efficacy assessment are listed in Table 2. Our primary objective is to explore the potential superiority of clonidine vs placebo in decreasing delirium duration and severity, measured by Memorial Delirium Assessment Scale (MDAS) [58] in patients diagnosed with delirium or subsyndromal delirium (according to Diagnostic and Statistical Manual of Mental Disorders, DSM-5 [59]). The primary endpoint, the trajectory of delirium, is the severity of delirium (measured by MDAS) over time.

**Table 2:** **List of endpoints and measurements for efficacy assessment**

Endpoint	Measurements for efficacy assessment
**Primary**	
Delirium trajectory	MDAS
**Secondary** (also with subanalyses for subsyndromal delirium and hypoactive/hyperactive/mixed delirium)	
Time-to-first delirium resolution	DSM-5
Incidence of “full-scale” delirium	DSM-5
Severity of delirium	MDAS, OSLA
Delirium subtype	MDAS, OSLA, DelApp
Use of “rescue medication”/additional drugs (as other sedatives, analgetics and antipsychotics)	Registration of use of all medication
Length of hospital stay	Registrations
Patient distress	Checklist of Nonverbal Pain Indicators
Cognitive function in follow-up after 4 months	MMSE-NR, Clock drawing test, Ten-words memory test, Trial making test A and B, IQCODE, CDR
Independence in follow-up after 4 months	Barthel ADL, NEADL
Pharmacokinetic response to clonidine	Serum drug concentrations
Pharmacodynamic response to clonidine	BP, HR, ECG, RASS, OSLA, symptoms of bradycardia, orthostatic hypotension or other side-effects
Biomarkers	Blood samples
Institutionalisation	Registrations
Survival	Registrations
**Safety**	
Side effects of clonidine/in-hospital complications	BP, HR, ECG, sedation (RASS, OSLA), and any symptoms of bradycardia, orthostatic hypotension or other side-effects

### Secondary endpoints

We will compare the actively treated group with the placebo group with respect to secondary endpoints shown in Table 2. The main secondary endpoint is the duration of delirium monitored daily by the DSM-5 diagnostic criteria. We will also study the feasibility of oral clonidine in a geriatric ward and effects of clonidine upon a variety of outcomes as a means to design a potentially more definite study later. We will do per protocol analyses and exposure-response analyses based on plasma-concentration.

## Methods and design

### Study design

**Figure 1:**
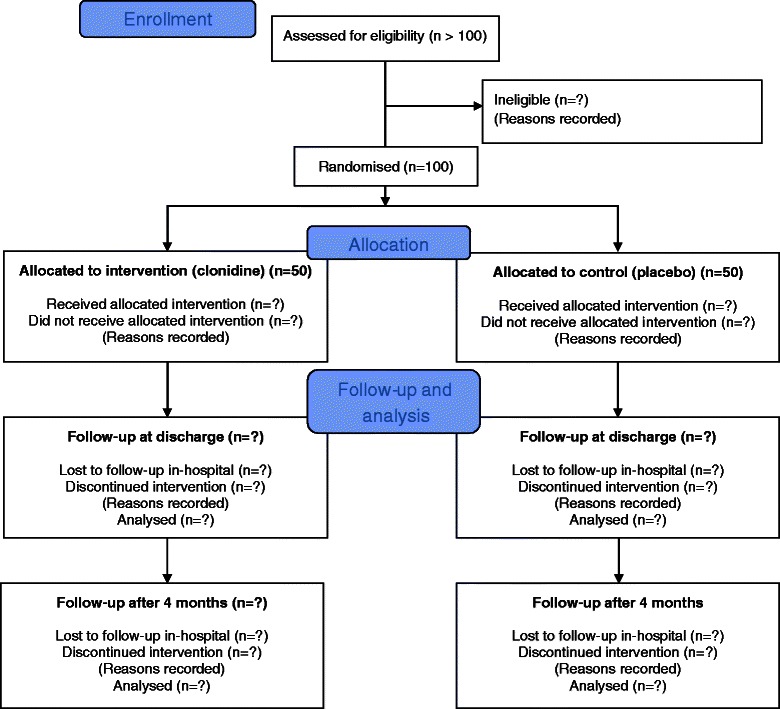
**CONSORT Study flow diagram.**

The Oslo Study of Clonidine in Elderly Patients with Delirium (LUCID) is a randomised, placebo-controlled, double-blinded, parallel group study with 4 month prospective follow-up (Figure 1). We aim to include 100 inpatients with delirium (or subsyndromal delirium) in an acute geriatric ward. Patients will be randomised to orally administrated clonidine or placebo until delirium free (by DSM-5 criteria) or no subsyndromal delirium for 2 days, or after a maximum of 7 days treatment. If the treating physician plan to discharge the patient (still having delirium/subsyndromal delirium), before 7 days, we will end the treatment 24 hours before discharge. Assessment of haemodynamics (blood pressure, heart rate and ECG) and of delirium will be performed daily until discharge, or a maximum of 7 days after end of treatment.

This study will be conducted in compliance with the Declaration of Helsinki and with ICH/Good Clinical Practice. Registration of patient data will be carried out in accordance with national personal data laws. The study design, protocol and informed consent procedures are approved by the Regional Medical Ethics Committee and the Norwegian Medicines Agency (EUDRACT number 2013-000815-26). The study is also registered at www.clinicaltrials.gov (NCT01956604).

### Study population

We will recruit patients >65 years of age from the acute geriatric ward at Oslo University Hospital. These patients are usually acutely admitted, arriving directly from the emergency department, with considerable multimorbidity and polypharmacy, with a high prevalence of infections, dehydration, acute cardiac problems, general medical problems, functional decline and delirium. They might be included at admission (prevalent delirium), or during the stay (incident delirium). Patients with prevalent delirium/subsyndromal delirium must be included within approximately 48 hours after admission and patients with incident delirium/subsyndromal delirium must be included within approximately 48 hours of symptom onset. All patients must have current delirium/subsyndromal delirium at the time of inclusion.

### Screening

Patients admitted to the study ward will go through a simple screening process (see Table 3). This screening is a combination of the Single Question in Delirium (SQiD) (asking one question to the patient's friend or relative:” Do you think (name) has been more confused in the last two weeks?”) [60], combined with two simple attention tests (reciting the days of the week and months of the year backwards). If any of these tests are positive, if the patient is drowsy, or if the nurse and/or the treating physician for other reasons suspects delirium, ascertainment of delirium or subsyndromal delirium will be performed.

**Table 3:** **Screening for delirium in patients at the acute geriatric ward**

		Yes	No
Screening	SQiD	□	□
	Drowsiness	□	□
	Cannot recite months backwards (unable to reach July)	□	□
	Cannot recite all the weekdays backwards	□	□
	Staff suspect delirium	□	□
Ascertainment of delirium or subsyndromal delirium	If Yes in any box, do diagnostic procedure (DSM-5), Table 4		
Inclusion	All DSM-5 criteria (delirium) or subsyndromal delirium → can be included		
	If the patient meets all the inclusion criteria, the patient will be included		

### Diagnosis of delirium and subsyndromal delirium

The diagnosis of delirium will be made by the study physicians according to DSM-5 criteria by using a standardised procedure (Table 4). Level of arousal will be assessed using the Richmond Agitation and Sedation Scale (RASS) [61] and the Observational Scale of Level of Arousal (OSLA) [62]. Attention will be evaluated using objective tests (Table 4) and observations by the examiner of the patient´s distractibility, comprehension and tendency to lose the thread of conversation. We will also use a smartphone (Android) software application, the “DelApp” [63], which incorporates an initial test of level of arousal followed by a test of attention. Acute change in the patient´s mental status, and fluctuation of any disturbance, will be ascertained through informant history from nursing staff and carers and also derived from clinical notes. Assessment of additional mental status disturbances will be performed by asking the patient a list of pre-defined questions (Table 4) in addition to information derived from nursing staff and clinical notes. All these assessments will be used in combination in relation to the DSM-5 criteria. The objective indicators (described above and in Table 4) will be supplemented by the assessor´s judgement regarding subjective features and a final diagnosis made.

**Table 4:** **Diagnostic algorithm for DSM-5 delirium**

DSM-5 criteria	Tests to be performed or information needed			DSM-5 criteria fullfilled?	
				YES	NO
A. Disturbance in attention (i.e., reduced ability to direct, focus, sustain, and shift attention) and awareness (reduced orientation to the environment).	**Evaluation**	**TEST**	**Cut off (definition of inattention)**		
	**Daily**	Digit span forward	Less than 5 forward		
	**Daily**	Digit span backward	Less than 3 backwards		
	**Daily**	SAVEAHAART	2 or more errors		
	**Initial diagnosis only**	Days of the week in reverse order	Any error		
	**Initial diagnosis only**	Months of the year in reverse order	Unable to reach July		
	**Initial diagnosis only**	Count backwards from 20 to 1	Any error		
	Observation (by the examiner):				
	Distractibility. Comprehension. Tendency to lose the tread of conversation				
	The “DelApp” [level of arousal test followed by counting of serially-presented lights. Cut-off 7/8 out of 10]				
B. The disturbance develops over a short period of time (usually hours to a few days), represents a change from baseline attention and awareness, and tends to fluctuate in severity during the course of a day.	Informant history from patient´s carers and nursing staff				
	Questions to carer/ nursing staff or derived from clinical notes:				
	Has there been a sudden change in the patient´s mental state?				
	Does the patient seem to be better at any period in the day compared to other times?				
	Has the level of consciousness been altered (drowsy/not interacting or agitated)?				
	Sleep-wake cycle disturbances?				
C. An additional disturbance in cognition (e.g., memory deficit, disorientation, language, visuospatial ability, or perception).	Questions to the patient:				
	Orientation to time, place and person				
	Recall (3 words)				
	Why are you in hospital? Will a stone float in water? Are there fish in the sea? (any error = disorganised thinking)				
	Questions to carer/ nursing staff or derived from clinical notes:				
	Has there been any…:				
	Perceptual disturbances? Sleep-wake cycle disturbances? Memory disturbances? Psychotic symptoms?				
	Psychomotor abnormalities?				
D. The disturbances in criteria A and C are not explained by another preexisting, established, or evolving neurocognitive disorder and do not occur in the context of a severely reduced level of arousal, such as coma.	Information from history/chart/clinical assessment				
E. There is evidence from the history, physical examination, or laboratory findings that the disturbance is a direct physiologic consequence of another medical condition, substance intoxication or withdrawal (i.e., because of a drug of abuse or to a medication), or exposure to a toxin or is because of multiple etiologies.	Information from history/chart/clinical assessment				
Delirium based on the tests and information above?	All DSM-5 criteria fulfilled			Yes □	No □
Subsyndromal delirium based on the tests and information above?	Defined as evidence of change, in addition to any one of these: (a) altered arousal, (b) attentional deficits, (c) other cognitive change, (d) delusions or hallucinations.			Yes □	No □
	Criteria D and E must be met.				

Subsyndromal delirium will be defined as evidence of change, in addition to any one of these: (a) altered arousal, (b) attentional deficits, (c) other cognitive change, (d) delusions or hallucinations. DSM-5 criteria D and E must be met. The severity of delirium will be assessed for all included patients by MDAS [58], based on the tests and information described above. We will also register the Confusion Assessment Method (CAM) [64] daily.

### Evaluating patient eligibility

According to the inclusion and exclusion criteria, ECG, creatinine/eGFR, body weight and blood pressure measurements are required. The final decision on eligibility will be agreed with the treating physician on the ward. Capacity will be assessed and informed consent (and/or) assent obtained (see below).

### Inclusion and exclusion criteria

Patients with delirium or subsyndromal delirium will be included in this study by the study physicians. We will include patients with chronic cognitive impairment or dementia as well as patients free from dementia. Nursing home patients are eligible. Patients must be > 65 years old, though the average age is expected to be more than 80 years. Further, the patient must be willing and able to receive the study medication.

**Inclusion criteria**

All of the following conditions must apply to the prospective patient at screening prior to receiving study agent:

- Patient > 65 years old admitted to the acute, medical, geriatric ward
- Delirium or subsyndromal delirium within the last 48 hours
- Signed informed consent from patient or relatives and expected cooperation of the patients for the treatment and follow up must be obtained and documented

**Exclusion criteria**

Patients will be excluded from the study if they meet any of the following criteria:

- Symptomatic bradycardia, bradycardia due to sick-sinus-syndrome, second- or third- degree AV-block (if not treated with pacemaker) or any other reason causing HR <50 bpm at time of inclusion [44].
- Symptomatic hypotension or orthostatic hypotension, or a systolic BP <120 at the time of inclusion
- Ischemic stroke within the last 3 months or critical peripheral ischemia
- Acute coronary syndrome, unstable or severe coronary heart disease (symptoms at minimal physical activity; NYHA 3 and 4) and moderate to severe heart failure (NYHA 3 and 4). (Acute coronary syndrome is defined according to international guidelines)
- A diagnosis of polyneuropathy, phaeochromocytoma or renal insufficiency (estimated GFR < 30 ml/min according to the MDRD formula) [44]
- Body weight <45 kg
- Considered as moribund on admission
- Unable to take oral medications
- Current use of tricyclic antidepressants, monoamine reuptake inhibitors or ciclosporin
- Previously included in this study
- Adverse reactions to clonidine or excipients (lactose, saccharose)
- Not speaking or reading Norwegian
- Any other condition as evaluated by the treating physician

### Informed consent and enrolment

Informed consent is a well-known challenge in studies of delirium [65,66], both due to the nature of delirium itself and the fact that people with underlying dementia are most at risk of developing delirium. Cognitively intact patients are included in this study on the basis of written, informed consent. We have developed a comprehensive information leaflet for cognitively intact patients and a simplified and shortened version for those who are partly or not at all competent to give their consent. For patients considered to lack capacity (due to severe delirium and/or dementia), but who are willing to take part, we will obtain proxy informed consent from a close relative. Consent to remain in the research will be obtained as soon as possible if capacity returns. Due to the importance of including patients as soon as possible after the diagnosis of delirium is made, the close relatives may give verbal consent (by phone) before randomisation and the written consent will be obtained as soon as possible after inclusion. We have good experience with this method from previous studies in our research group [67].

### Randomisation and blinding

The randomisation is based on computer-generated random numbers, and will be carried out by a statistician. The randomisation schedule will be distributed to the producer of the study medication, and capsules made accordingly. The randomisation will be stratified with respect to whether or not the patient was admitted from a nursing home, in order to balance the groups with respect to pre-admission cognitive decline, independence and comorbidity, all important prognostic factors.

### Study medication

Each capsule (CAPSUGEL) will contain either 75 μg Catapresan (clonidine hydrochloride) or 75 μg placebo, and will be produced and labeled by Kragerø tablettproduksjon A/S. The capsules containing active medication and placebo will look identical. If other medications are indicated for the treatment of delirium, the treating physician will prescribe this as “standard care”.

### Monitoring and safety

BP and HR will be monitored 3 hours after each loading dose (day 1) and then just before every dose. If there are signs of significant hypotension (systolic BP <100 mmHg) or bradycardia (heart rate <50 beats/min), further monitoring or treatment will be considered individually. During the whole treatment period blood pressure and heart rate will be assessed twice per day. ECG, serum creatinine, blood glucose and a clinical assessment by a physician (hydration, side-effects, RASS [61] and OSLA [62]) will be performed daily. Orthostatic BPtests will be performed during the hospital stay at day 5, 6 or 7 at 1100 (approximately 3 hours after administration of study drug).

For all patients, drug concentration (mean concentration just before intake and Cmax 3 hours after intake) will be measured at day 5, 6 or 7. The pharmacokinetics of clonidine varies inter-individually, possibly influencing individual treatment responses. But due to the short treatment period, measurement of drug concentration for individual drug modification is not feasible.

### Data collection

Demographic data, medical history, information regarding the acute underlying medical disease(s), drug use and proxy information will be collected during the hospital stay. Once included in the study, patients will be visited daily for efficacy and safety evaluations by the study physicians (geriatricians) or by the consultant on-call during the weekend.

We will assess pre-existent functional and cognitive status by asking the patient’s primary caregiver (the best available source) to complete questionnaires to assess the patient´s functional and cognitive state two weeks prior to hospital admission. Functional status will be assessed using the Barthel ADL Index [69] and the Nottingham Extended ADL Index (NEADL) [70]. To ascertain prior long-term cognitive decline we will use the Informant Questionnaire on Cognitive Decline in the Elderly (IQCODE) [71] and the Clinical Dementia Rating Scale (CDR) [72]. The Mini-Mental State Evaluation – Norwegian version (MMSE-NR) [73,74] will be performed at baseline and at discharge for the purposes of general cognitive screening. The Cornell Depression Scale [75,76] is based on proxy information and will be used to evaluate the degree of depressive symptoms.

Grip strength of the dominant hand will be measured using hand-held dynamometry once during the hospital stay and at follow-up after 4 months. The severity and number of comorbidities will be scored using the Cumulative Illness Rating Scale (CIRS) [77]. The level of physiological disturbance will be assessed by using Acute Physiology and Chronic Health Evaluation II (APACHE II) [78], the version utilising venous bicarbonate rather than arterial blood gases. Body Mass Index is registered as a marker of nutritional status.

We will use the algorithm described in the section above and in Table 4 to diagnose delirium (or subsyndromal delirium) according to the DSM-5 criteria.

We will also collect data about patient distress, using items from The Checklist of Nonverbal Pain Indicators (CNPI) [79]. Possible and suggestive causes of delirium are registered in each case at discharge.

### Laboratory tests and blood sample procedures

Data from routine blood samples taken at admission will be recorded (including Erythrocyte Sedimentation Rate, Haemoglobin, Leukocytes, Creatinine, Electrolytes, C-reactive protein, Albumin, Thyroid stimulating hormone and free Thyroxine levels).

All patients will have blood drawn for drug concentration levels at day 5, 6 or 7 (just before and 3 hours after intake of study medication), to be able to compare possible effects and side-effects to the actual plasma-concentration at the end of the trial.

Our study population is expected to have significant acute and chronic comorbidity. In this aspect, looking for new biomarkers is challenging, as we do not have a delirium-free control group. We will however take blood samples (serum and plasma) for biomarkers at inclusion, at mid-stay (day 3–5) and at discharge, to explore markers already known to be associated with delirium. Interesting biomarker candidates include S100B [80,81], neopterin [82], IGF-1 [83], MMP-9, protein C, sTNFR1 [84]. The serum and plasma will be stored in a biobank freezer at Oslo University Hospital together with blood stored in EDTA tubes for possible DNA-analyses.

### Follow-up assessments

We will phone the patient or care-taker by phone one week after the end of the treatment to consider possible side-effects/ rebound effects or relapse of the delirium. No physical examination or psychometric tests will be performed. We will register the level of care, and whether the patient has been discharged home or to an institution.

Four months after discharge, a home visit will be done to perform cognitive tests (see Table 2). Based on proxy information, we will evaluate each patient´s level of independence and functional and cognitive status. We will assess the presence of persistent delirium (according to DSM-5 criteria) and subsyndromal delirium and perform the MDAS. Grip strength will be measured. Level of care, any readmissions to hospital and cumulative mortality will be registered.. Causes of death will be ascertained from the Cause of Death Register.

### Criteria for patient discontinuation

Patients may be discontinued from study treatment and assessments for several reasons. These include: omission of more than 3 following dosages, voluntary discontinuation by the patient, safety reasons as judged by the study physician or treating physician, or significant non-compliance with the protocol as judged by the study physician. Any patient withdrawn from the study will be included in the statistical intention-to-treat analysis. If possible, a final assessment shall be made (end of study visit) and the reason for discontinuation shall be recorded. Progression of delirium is not a discontinuation criterion, as the trajectories are variable and impossible to predict. Progression of delirium may however make the patient unwilling to further participate. If any of the exclusion criteria appear during the treatment, treatment will be discontinued. (Except changes in blood pressure and heart rate, which will be managed as described in the dosage plan).

### Statistical analysis

The primary endpoint is the repeated measurements of MDAS over time. Differences in the MDAS trajectories between the treatment groups will be analysed by a mixed linear model. In addition we will, as a secondary endpoint, compare the time to resolution of delirium as measured by DSM-5. The Kaplan Meier method and the logrank test will be applied. In addition a Cox proportional hazards model will be applied to estimate hazard ratios. The additional different secondary endpoints will be analysed by t-tests when variables are continuous and by chi-square tests when variables are categorical. Patient survival will be compared between groups by the logrank test and Cox proportional hazards model.

For the comparison of the MDAS trajectories no adjustment for multiplicity will be applied. If a statistically significant difference between the MDAS trajectories is demonstrated, analyses of secondary endpoints will be performed without any formal adjustment for multiplicity. If any conclusion on efficacy is to be drawn based on a secondary endpoint only, a simple Bonferroni adjustment (dividing the 5% level with the actual number of tests performed) will be applied. If the use of additional antipsychotics differs between treatment groups, the amount calculated as haloperidol equivalents [68] will be included in the statistical model.

### Sample size, statistical power and statistical analysis

Based on the expected number of patients fulfilling the inclusion criteria within 36 months a sample size of n = 100 was chosen. Inclusion of 100 patients would lead to a power of only 71% to detect an absolute treatment difference of 25% in proportion recovered according to DSM-% (40% vs 65%). Correspondingly, using MDAS at one single point in time as the response measure would lead to approximately 80% power assuming a standard deviation of 9 and a difference in mean MDAS score of 5. Thus, analyzing treatment efficacy at only one point in time would lead to low power. An analysis of repeated measurements will reduce random variability and thereby increase power. The primary analysis is therefore a mixed linear model taking all MDAS measurements into account.

The expected efficiency gain is difficult to estimate precisely, but assuming a standard deviation of 9 on the MDAS and a correlation between measurements of r = 0.5, the power would be around 95% to detect a mean MDAS difference of 5 or a power of 80% to detect a mean difference of 3.5 between groups.

## Discussion

Delirium is a severe and common condition among acute hospitalised elderly patients, and the options for pharmacological treatment are sparse. To our knowledge, no randomised placebo controlled trial investigating treatment of delirium with clonidine or other alpha-2-adrenoreceptor agonists has yet been done in non-critically ill medical patients. It has been recommended [85,86] that future studies on pharmacological treatment of delirium should have less restrictive exclusion criteria, in order to avoid low external validity. Further, future studies should ensure stratification according to known underlying risk factors for delirium. The trials should then have a true placebo arm and use validated instruments for delirium assessments. We have planned this trial according to these guidelines.

The results of our study will be more clinically relevant if the medication is easy and safe to administer to patients in a general ward, without the need for invasive monitoring. Frail and demented patients are more prone to develop delirium, and the treatment regimen should be feasible for this large group of patients. Thus, it is important that the exclusion criteria are not too strict, and we will therefore include patients with dementia and patients from nursing homes in this trial.

Delirium is a clinical diagnosis, based on history, information from proxies, mental status examination and evaluation of an underlying medical cause. There is no single simple test, neither psychometric nor physiological, to ascertain delirium. We have described the procedure that we will use to make the final diagnosis according to the DSM-5 criteria. All assessments of delirium will be made by the investigators (geriatricians trained in delirium evaluation) during weekdays, and by geriatricians on call in the weekends.

The primary outcome in this study is the trajectory of delirium, measured daily by MDAS. Due to features common in studies of delirium (small sample sizes, sample attrition, fluctuation course of the disease and spontaneous recovery), techniques that use all available data and take a global (as opposed to end-of-trial) perspective are preferred [87]. Using longitudinal trajectories as the main outcome will almost always have greater power than end-of-trial analyses [87-89], as well as reducing the problem with missing data.

It is important to determine whether clonidine will have a differential impact on hyperactive versus hypoactive subtypes of delirium, or even only on certain features of delirium (e.g. inattention). The sample size of this study is too small to give conclusive results regarding secondary endpoints. However, these secondary objectives are exploratory and may generate new hypotheses and give direction for future studies.

Any positive results of clonidine on delirium symptoms will also contribute to the discussion about the underlying pathophysiology and the role of the autonomic nervous system in delirium. Studies of dexmedetomidine have showed promising results with respect to delirium duration and severity in the intensive care unit patients, but it is still uncertain weather this is an opioid- and benzodiazepine-sparing effect alone or if dexmedetomidine has truly delirium modulating effects [36,37]. Any positive effect of clonidine compared to placebo may indicate that the sympathetic nervous system and related stress systems are involved in the underlying pathophysiology of delirium.

## Conclusion

LUCID will contribute to knowledge about the pharmacological treatment of delirium in the elderly, and may also shed light on relevant pathophysiological hypotheses.

## Abbreviations

ADL: Activities of daily living
AV- block: Atrioventricular block
BID: Twice daily (bis in die)
BP: Blood Pressure
BPM: Beats per minute
CAM: Confusion assessment method
CDR: Clinical dementia rating scale
CFS: Chronic fatigue syndrome
CIRS: Cumulative illness rating scale
Cmax: Maximum plasma concentration
CNS: Central Nervous System
DSM-5: Diagnostic and statistical manual of mental disorders, fifth edition
ECG: Electrocardiogram
GFR: Glomerular filtration rate
HR: Heart rate
ICH: International conference on harmonization
ICU: Intensive care unit
IMP: Investigational medicinal product
IQCODE: Informant questionnaire on cognitive decline in the elderly
MAP: Mean Arterial Pressure
MDAS: Memorial delirium assessment scale
MDRD: Modification of diet in renal disease
MMSE-NR: Mini mental state evaluation – Norwegian version
NYHA: New York Heart Association (functional classification of heart disease)
OSLA: Observational scale of level of arousal
RASS: Richmond agitation sedation scale
RCT: Randomised controlled trial
SQID: Single question in delirium
